# Multimorbid Patient Experiences With Primary Care at Community Health Centers in Shanghai, China

**DOI:** 10.3389/fpubh.2021.606188

**Published:** 2021-05-31

**Authors:** Hua Jin, Zhaoxin Wang, Leiyu Shi, Chen Chen, Yongyan Huo, Wuquan Huang, Yi Zhang, Yuan Lu, Xuhua Ge, Jianwei Shi, Dehua Yu

**Affiliations:** ^1^Department of General Practice, Yangpu Hospital, School of Medicine, Tongji University, Shanghai, China; ^2^Shanghai General Practice and Community Health Development Research Center, Shanghai, China; ^3^School of Public Health, Shanghai Jiaotong University School of Medicine, Shanghai, China; ^4^Department of Health Policy and Management, Primary Care Policy Center, Johns Hopkins University, Baltimore, MD, United States; ^5^Shanghai Jing'an District Jiangning Road Community Health Service Center, Shanghai, China; ^6^Shanghai Jiading District Anting Town Huangdu Community Health Service Center, Shanghai, China; ^7^Shanghai Jiading District Jiading Town Community Health Service Center, Shanghai, China; ^8^Shanghai Xuhui District Fenglin Community Health Service Center, Shanghai, China

**Keywords:** multimorbidity, primary care, quality, PCAT, China

## Abstract

**Objective:** Primary care in China is facing mounting challenges with multimorbidity as the aging population grows. Knowing how patients experience primary care may highlight the deficiencies of the care system and guide health system reform. The purpose of this study was to compare the quality of primary care experienced by patients with and without multimorbidity at community health centers (CHCs) in Shanghai, China and to examine the factors influencing these experiences.

**Methods:** A cross-sectional survey was conducted from August to December 2019 using the validated Chinese Primary Care Assessment Tool-Adult Edition (PCAT-AE). ANOVA was performed to compare the overall and domain-specific quality of primary care for patients with and without multimorbidity. Multivariate linear regressions were used to assess the factors associated with primary care quality while controlling for patients' sociodemographic and healthcare characteristics.

**Results:** From 2,404 completed questionnaires, patients with multimorbidity reported higher PCAT scores in the domains of first contact-utilization (3.54 ± 0.55 vs. 3.48 ± 0.56, *P* < 0.01), accessibility (2.93 ± 0.49 vs. 2.86 ± 0.47, *P* < 0.001), and ongoing care (3.20 ± 0.39 vs. 3.14 ± 0.43, *P* < 0.001), while reporting lower scores in coordination (information system) (2.72 ± 0.41 vs. 2.79 ± 0.35, *P* < 0.001) and family-centeredness (3.23 ± 0.63 vs. 3.30 ± 0.64, *P* < 0.01). Multimorbidity (ß = 0.355, *P* < 0.01), education level (ß = 0.826, *P* < 0.01), district (suburb: ß = 1.475, *P* < 0.001), and self-perceived good health status (ß = 0.337, *P* < 0.05) were associated with better patient experiences in primary care. Patients between the age 61 and 70 (ß = −0.623, *P* < 0.001; >70 years: ß = −0.573, *P* < 0.01), with a monthly household income ≥6,000 RMB (ß = −1.385, *P* < 0.001) and with more than 20 outpatient visits the previous year (ß = −1.883, *P* < 0.001) reported lower total PCAT scores.

**Conclusion:** The findings of our study suggest that CHCs in China have contributed to better primary care experiences for patients with multimorbidity in certain quality domains, including first contact-utilization, accessibility, and ongoing care. However, there is still room for improvement in care coordination and family-centeredness.

## Introduction

As population ages, addressing the resulting increase in multimorbid patients (patients having two or more chronic diseases at the same time) (1) has become a priority for global health and a necessary consideration for medical resource consumption. As of 2016, it was estimated that between 20 and 30% of the global population were experiencing multimorbidity, with much higher rates of 66–90% of people over 65 (2). Especially in a primary care setting, multimorbidity is becoming the norm rather than exception (3, 4). The prevalence of multimorbidity in primary care patients has ranged from 1 to 90% in different studies (5–7). In China, a sample survey study in Guangdong Province found that 11.1% of the population had multimorbidity (8). Among older inpatients, however, the multimorbidity rate was 63.7% (9).

Multimorbidity is not only associated with an increased burden on medical resources, but also with more complex needs in medical care. Research has shown that patients with multimorbidity have an increased risk of mortality, functional decline, disability, poor quality of life, and harmful medication-related events (10–12). A qualitative study of healthcare providers supporting patients with multiple chronic conditions found that providers overwhelmingly supported increased emphasis on patient-centered, comprehensive, and integrated care (13). Specifically, physicians need to thoroughly understand the patient's situation, emphasize disease prevention, and enhance collaboration with specialists and medical institutions at different levels (14). As a result, primary care has been highly recommended in international research as the most suitable service model to deliver care to patients with multimorbidity (15). Primary care provides integrated, accessible healthcare services by clinicians who can address a large range of personal healthcare needs, develop a sustained partnership with patients, and practice in the context of family and community (16, 17). Studies have shown that patient-oriented interventions in primary care may lead to better health outcomes, including improvements in patients' medication adherence and benefits to patient and provider behavior (18, 19).

Starting in 2009, China launched a new round of medical care reform with considerable emphasis on primary care and care for those with serious conditions. The government invested substantial financial resources and issued policies to strengthen primary care and attract a wide range of patients. These policies included improved training for general practitioners (GPs) and the integration of clinical care with basic public health services for community residents (including maternity, child, and elderly care, disease management of diabetes, hypertension, and mental illnesses, health education, immunizations, and communicable disease prevention and reporting) (20, 21). Previous studies have demonstrated the impact of policy and structural change on the quality of primary care nationwide (22–24). A study in Shanghai found that primary care policy promoted long-term provider-patient relationships and coordinated service with hospitals, while capitation payments for the GP team may have contributed to an improvement in care quality (25). Hypertensive patients in Shanghai also experienced better information coordination than hypertensive patients in Shenzhen, another one of the most developed cities in southern China (26).

However, despite notable progress, there remains a large gap between what patients and providers need, and the quality and effectiveness of care delivered in China. Whether CHCs in Shanghai are prepared to take on the significant multimorbidity burden of an aging population has yet to be assessed. Our study examined the quality of primary care experiences for multimorbid patients in Shanghai, China using the Primary Care Assessment Tool (PCAT). To the best of our knowledge, this was the first study to assess primary care quality from the perspective of patients with multimorbidity in China. The objective of this study was to gauge the quality of multimorbid patient primary care experiences in Shanghai in order to identify areas for improvement of care quality in China.

## Methods

### Study Setting

This was a cross-sectional survey conducted in Shanghai, China, a city with 14.5 million registered residents and 9.8 million non-registered residents as of 2019. Shanghai is one of the most developed cities in China; its GDP per capita was the second highest in China (157,300 RMB) (27). As one of the pilot cities to implement healthcare reform, Shanghai developed the “1+1+1” (one CHC + one regional secondary hospital + one tertiary hospital) family contract policy in 2015 to encourage residents to utilize CHC services. The GP team provides regular prescriptions, referrals, chronic disease management, public health services, nursing, and other community-based medical services. By the end of 2018, there were 6.66 million Shanghai residents participating in the contract policy with a sign-up rate of 30%. Among vulnerable populations, including those over 65 years of age, pregnant, or disabled, the sign-up rate was over 80% (28, 29).

We used a multistage sampling method in this study. In the first stage, all 244 CHCs in Shanghai were classified into two groups according to their quality performance scores as assessed by the 2019 Annual Report of Health Center General Practice Quality Performance (30) (i.e., those ranked in the upper 50th percentile and those ranked in the lower 50th percentile) so as to include both higher and lower performing CHCs in the study. In the second stage, all CHCs were classified into three clusters according to their geographic location: urban, suburban or rural. We used computer-generated random numbers to choose two CHCs from each cluster. In the third stage, we contacted the randomly selected CHCs with the help of local government officials and community residential committees to ask if they would like to participate in our survey. In total, all 12 selected CHCs agreed to take part in our study.

Patients (aged 40 years and above) who had visited the given CHC at least twice within the past 6 months were invited to participate in the survey. Those who had trouble understanding the questionnaire (i.e., patients with cognitive dysfunction, hearing or language disorders) and those who were in poor physical condition and could not complete the survey were excluded. The target sample size was calculated based on the proportion of patients who responded favorably to PCAT questions first obtained through a pilot (i.e., 85%) and then using 5% as the margin of error. A minimum sample size of 200 patients per CHC was required for selecting patients from the target population (i.e., CHC-contracted residents above 40 years of age).

### Data Collection

The survey was conducted from August 2019 to December 2019. Data were collected through face-to-face interviews and questionnaires were administered by investigators in the study. To reduce interviewer bias, we conducted training with all interviewers prior to actual data collection so that questions and answers were provided consistently. We also conducted a pre-test to allow interviewers to practice with actual patients while monitored. In the early phase of the study, all interviewers were supervised during the actual interview session until proficient in administering the questionnaire.

### Measurement

We used the Primary Care Assessment Tool-Adult Edition (PCAT-AE) to assess participants' experiences with primary care. This tool was designed by Professors Barbara Starfield and Leiyu Shi of the Primary Care Policy Center at Johns Hopkins University initially for application in the US and was provided to researchers free of charge. It measures four exclusive attributes of primary care: first contact, longitudinality, comprehensiveness, and coordination, as well as three supplemental attributes: family-centeredness, community orientation, and cultural competence (31). PCAT has gradually been adapted in many other regions and countries (32–35) including China (36). The Johns Hopkins team developed a Chinese version and tested it with adult sample subjects from the southern part of China (Guangzhou Province) and the western part of China (Tibet Province). The sample tests verified the Chinese PCAT as reliable and valid (37–39). In this study, we used the Chinese version of the PCAT with the developers' consent.

The PCAT-AE survey includes 87 items to assess participants' primary care experience across 10 domains: first contact-utilization, first contact-accessibility, ongoing care, coordination (referral system), coordination (information system), comprehensiveness (services available), comprehensiveness (services provided), community orientation, family-centeredness, and cultural competence. First contact-utilization addresses general routine examinations as well as first diagnosis of new health problems and other content; first contact-accessibility refers to business hours, receiving same-day medical treatment, telephone consultations, evening home visits, appointments for general physical examinations, waiting time, difficulty obtaining medical treatment, expectation value, etc.; coordination (referral system) pertains to referral services between primary care and specialists; coordination (information system) consists primarily of previous medical records; the comprehensiveness of services available and provided includes medical services provided by the CHC; family-centeredness refers to family involvement in medical procedures and consideration of family history in care; community orientation includes family visits, an understanding of regional health issues, and listening to patients and family; and cultural competence refers to the ability of CHC treatment to be recommended to relatives and friends.

A patient's experience was measured by a five-point Likert-type scale in which 1 = definitely not, 2 = probably not, 3 = probably, 4 = definitely, and 9 = not sure/don't know (when calculating, 9 was replaced with a score of 2.5 based on the PCAT manual). Scores for each domain were derived from the average score of all items within the domain. Higher scores represent better patient primary care experience (40). The mean PCAT-AE score was calculated by taking the mean of all 10 domain scores and reflects an overall measure of the quality of a patient's primary care experience.

In addition, items pertaining to socio-demographic characteristics were included in the questionnaire, such as gender, age, marital status, employment status, education level, average monthly family income, region, and health insurance. Items identifying health service utilization were also included (i.e., the frequency of CHC visits, the number of CHC visits in the previous year, the number of hospitalizations in the previous year, self-perceived health status, physical or mental disease lasting over 1 year, and number of chronic diseases). Multimorbidity was defined as suffering from two or more than two chronic diseases at the same time. Chronic diseases included in multimorbidity analysis were based on the list of conditions for measuring multimorbidity in the current literature (41), for example, diabetes, hypertension, chronic obstructive pulmonary disease (COPD), arthritis, heart disease (heart failure and myocardial infarction), etc.

### Analysis

We divided participants into two groups (with and without multimorbidity) according to the number of chronic diseases they had. Normal distribution was assessed with a normal Q–Q plot. We first compared the groups' socio-demographic characteristics and healthcare utilizations using Chi-squared tests. Next, total primary care attributes between the two groups were examined using independent 2-sample *t*-tests. Multiple linear regression models were then constructed to compare the two groups after controlling for socio-demographic characteristics (sex, age, marital status, educational level, employment, household income, health insurance, and region) and healthcare measures (frequency and number of CHC visits, number of times seeking outpatient services in a CHC, number of hospitalizations, health status, physical or mental disease lasting over 1 year, and multimorbidity). All independent variables were tested for collinearity, but no collinearity was present. There were only 851 contracted patients who reported experiencing a referral, therefore, coordination domain (referral system) was excluded from total PCAT scores calculation when conducting multiple linear regression. For all tests conducted in the study, a *p* < 0.05 was assigned as the level of statistical significance. All analyses were performed using SPSS 20.0.

## Results

### Demographic Characteristics of Participants

Table 1 shows the demographic, socio-economic and health status characteristics of participants in the study. A total of 2,404 participants were surveyed, and 1,303 of these patients suffered from multimorbidity. As shown in Table 1, 54.78% of the respondents were female and 47.80% were between age 61–70. Most participants were married (98.88%) and unemployed/retired (63.85%). Most individuals' highest education level was either primary school or below (37.53%) or junior school (35.36%), and 34.73% had an average monthly family income of ≤ 3,000 RMB. 82.53% had health insurance. Patient distribution in the urban area (31.91%) was similar to that of suburban and rural areas. In terms of healthcare utilization, 72.8% of patients reported visiting a CHC more than once per month. The proportion seeking outpatient services <10 times in the previous year was 33.32%, followed by >20 times (27.08%) and 10–15 times (26.04%). 87.67% of respondents had not experienced inpatient hospitalization in the previous year and 57.45% reported poor/fair health status.

**Table 1:** Participant characteristics in the study (*N* = 2,404).

**Variable**	**Group**	**Total (*****n*** **=** **2,404)**		**Without multimorbidity (*****n*** **=** **1,303)**		**With multimorbidity (*****n*** **=** **1,101)**		**Chi-square**	***P*-value**
		***N***	**%**	***N***	**%**	***N***	**%**		
**Sociodemographic characteristics**									
Gender	Male	1,087	45.22	587	45.05	500	45.41	0.032	0.858
	Female	1,317	54.78	716	54.95	601	54.59		
Age (year)	≤ 60	504	20.97	360	27.63	144	13.08	70.647	<0.0001
	61–70	1,149	47.8	596	45.74	553	50.23		
	≥71	751	31.24	347	26.63	404	36.69		
Marital Status	Married	2,377	98.88	1,283	98.47	1,094	99.36	4.344	0.037
	Unmarried	27	1.12	20	1.53	7	0.64		
Employment status	Employed	869	36.15	515	39.52	354	32.15	14.05	<0.0001
	Unemployed/retired	1,535	63.85	788	60.48	747	67.85		
Education (missing = 6)	Primary school or below	900	37.53	443	34.00	457	41.51	27.032	<0.0001
	Junior school	848	35.36	453	34.77	395	35.88		
	Senior high school	450	18.77	272	20.87	178	16.17		
	College or above	200	8.34	132	10.13	68	6.18		
Average monthly household	≤ 3,000	835	34.73	462	35.46	373	33.88	2.673	0.102
income (RMB)	3,001–4,000	515	21.42	302	23.18	213	19.35		
	4,001–6,000	503	20.92	255	19.57	248	22.52		
	≥ 6,000	305	12.69	148	11.36	157	14.26		
	Not sure	246	10.23	136	10.44	110	9.99		
Medical insurance	No	420	17.47	215	16.50	205	18.62	1.859	0.173
	Yes	1,984	82.53	1,088	83.50	896	81.38		
District	Urban	767	31.91	439	33.69	328	29.79	9.974	0.007
	Suburb	819	34.07	408	31.31	411	37.33		
	Rural	818	34.03	456	35.00	362	32.88		
**Health care utilization**									
Frequency of seeking health service in CHC	More than once per month	1,750	72.8	801	61.47	949	86.19	160.711	<0.0001
	Every 1–3 months	311	12.94	230	17.65	81	7.36		
	More than every 3 months	245	10.19	194	14.89	51	4.63		
	Not sure	98	4.08	78	5.99	20	1.82		
Times of outpatient visiting	≤ 10	801	33.32	593	45.51	208	18.89	202.43	<0.0001
in the previous year	10–14	626	26.04	320	24.56	306	27.79		
	15–20	326	13.56	155	11.90	171	15.53		
	>20	651	27.08	235	18.04	416	37.78		
Times of hospitalization in	0	2,084	87.67	1,187	91.10	897	81.47	51.949	<0.0001
the previous year	1	231	9.72	93	7.14	138	12.53		
(missing = 27)	≥2	62	2.61	13	1.00	49	4.45		
Self-percived health status	Fair/poor	1,381	57.45	635	48.73	746	67.76	88.339	<0.0001
	Good/very good	1,023	42.55	668	51.27	355	32.24		
Physical or mental disease	Yes	527	21.92	217	16.65	310	28.16	31.505	<0.0001
lasting over 1 year	No	1,698	70.63	986	75.67	712	64.67		
	Not sure	179	7.45	100	7.67	79	7.18		

Compared to respondents without multimorbidity, multimorbid patients were more likely to be older (age 61–70), employed, and have higher levels of education. There was a higher proportion of frequency of hospital visiting, CHC visiting, and inpatient hospitalization in the multimorbid group. These patients also reported poorer self-perceived health and a higher proportion of physical or mental disease lasting over 1 year.

### Primary Care Attributes

Table 2 shows comparisons of PCAT scores by participant characteristics. In this survey, the total score of the PCAT-AE was 30.84 ± 3.30. Among the 10 domains, first contact-utilization had the highest average score (3.51 ± 0.56), followed by family-centeredness (3.27 ± 0.63), comprehensiveness (services provided) (3.26 ± 0.54), coordination (referral system) (3.22 ± 0.58), comprehensiveness (services available) (3.20 ± 0.57), cultural competence (3.17 ± 0.64), ongoing care (3.16 ± 0.41), community orientation (3.11 ± 0.60), and first contact-accessibility (2.89 ± 0.48). The average score of coordination (information system) was lowest (2.76 ± 0.38).

**Table 2:** Comparison of PCAT scores by participants' characteristics.

**Variable**	**Group**	**First contact (utilization)**	**First contact (accessibility)**	**Ongoing care**	**Coordination (referral system)**	**Coordination (information system)**	**Comprehensiveness (available)**	**Comprehensiveness (provided)**	**Family centeredness**	**Community orientation**	**Culturally competent**	**Total**
		**Mean (SE)**	**Mean (SE)**	**Mean (SE)**	**Mean (SE)**	**Mean (SE)**	**Mean (SE)**	**Mean (SE)**	**Mean (SE)**	**Mean (SE)**	**Mean (SE)**	**Mean (SE)**
**Socio-demographic characteristics**											
Gender	Male	3.51 (0.54)	2.92 (0.48)	3.16 (0.41)	3.23 (0.56)	2.77 (0.36)	3.19 (0.57)	3.26 (0.54)	3.27 (0.63)	3.10 (0.60)	3.15 (0.64)	30.85 (3.33)
	Female	3.51 (0.57)	2.87 (0.47)	3.17 (0.42)	3.22 (0.60)	2.74 (0.39)	3.21 (0.57)	3.26 (0.53)	3.27 (0.64)	3.12 (0.61)	3.18 (0.63)	30.83 (3.28)
Age (year)	≤ 60	3.53 (0.54)	2.92 (0.44)	3.22 (0.39)^***^	3.19 (0.56)	2.80 (0.34)^*^	3.28 (0.56)^***^	3.34 (0.49)^***^	3.36 (0.58)^***^	3.21 (0.59)^***^	3.23 (0.62)^*^	31.45 (3.20)^***^
	61–70	3.5 (0.56)	2.90 (0.5)	3.14 (0.43)	3.17 (0.61)	2.75 (0.39)	3.19 (0.58)	3.23 (0.54)	3.24 (0.67)	3.07 (0.63)	3.15 (0.65)	30.68 (3.38)
	≥71	3.51 (0.56)	2.86 (0.47)	3.16 (0.39)	3.31 (0.54)^***^	2.74 (0.39)	3.15 (0.56)	3.24 (0.55)	3.26 (0.60)	3.11 (0.57)	3.15 (0.62)	30.68 (3.21)
Marital status	Married	3.51 (0.56)	2.89 (0.48)	3.16 (0.41)	3.22 (0.58)	2.76 (0.38)	3.20 (0.57)	3.26 (0.54)	3.27 (0.64)	3.11 (0.60)	3.17 (0.64)	30.85 (3.30)
	Unmarried	3.42 (0.64)	2.77 (0.71)	3.17 (0.36)	3.45 (0.34)	2.69 (0.44)	3.06 (0.73)	3.19 (0.58)	3.34 (0.46)	3.04 (0.71)	3.23 (0.58)	30.38 (3.68)
Employment status	Employed	3.54 (0.49)	3.00 (0.47)^***^	3.14 (0.40)	3.24 (0.50)	2.74 (0.39)	3.17 (0.54)	3.23 (0.54)	3.24 (0.56)	3.14 (0.54)^*^	3.05 (0.65)	30.72 (3.17)
	Unemployed/ retired	3.50 (0.59)	2.83 (0.47)	3.18 (0.42)^*^	3.21 (0.62)	2.77 (0.37)	3.22 (0.59)^*^	3.27 (0.53)^*^	3.29 (0.67)	3.09 (0.63)	3.23 (0.62)^***^	30.91 (3.37)
Education	Primary school or below	3.62 (0.52)^***^	2.99 (0.43)^***^	3.17 (0.46)	3.25 (0.52)	2.72 (0.40)	3.29 (0.51)^***^	3.30 (0.54)	3.23 (0.68)	3.16 (0.60)	3.20 (0.68)	31.17 (3.51)
	Junior school	3.45 (0.56)	2.88 (0.49)	3.17 (0.37)	3.20 (0.56)	2.77 (0.36)	3.18 (0.60)	3.21 (0.54)	3.29 (0.58)	3.08 (0.58)	3.11 (0.58)	30.67 (3.05)
	Senior high school	3.41 (0.58)	2.73 (0.47)	3.10 (0.40)	3.15 (0.69)	2.78 (0.37)	3.05 (0.63)	3.22 (0.51)	3.24 (0.63)	3.01 (0.62)	3.18 (0.63)	30.25 (3.17)
	College or above	3.51 (0.61)	2.82 (0.55)	3.28 (0.37)^***^	3.41 (0.54)^*^	2.80 (0.32)^***^	3.21 (0.49)	3.39 (0.51)^***^	3.40 (0.66)^***^	3.24 (0.60)	3.24 (0.69)	31.40 (3.41)^***^
Average monthly household income (RMB)	≤ 3,000	3.56 (0.55)^***^	3.00 (0.46)^*^	3.13 (0.48)	3.29 (0.50)	2.78 (0.37)	3.23 (0.57)	3.26 (0.56)	3.21 (0.67)	3.16 (0.60)	3.08 (0.66)	30.91 (3.61)
	3,001–4,000	3.44 (0.57)	2.86 (0.46)	3.23 (0.34)^*^	3.41 (0.45)^***^	2.82 (0.32)^***^	3.30 (0.48)^***^	3.33 (0.50)	3.42 (0.50)^***^	3.20 (0.50)^***^	3.22 (0.56)	31.37 (2.91)^***^
	4,001–6,000	3.53 (0.55)	2.80 (0.49)	3.21 (0.39)	3.30 (0.53)	2.73 (0.39)	3.26 (0.52)	3.33 (0.54)^***^	3.36 (0.65)	3.19 (0.60)	3.21 (0.65)	31.15 (3.46)
	≥ 6,000	3.49 (0.54)	2.88 (0.51)	3.20 (0.38)	3.06 (0.66)	2.69 (0.43)	3.11 (0.63)	3.21 (0.48)	3.10 (0.66)	3.00 (0.62)	3.23 (0.68)^***^	30.32 (2.78)
	Not sure	3.49 (0.58)	2.78 (0.44)	3.01 (0.35)	2.89 (0.70)	2.70 (0.42)	2.88 (0.67)	3.02 (0.50)	3.18 (0.62)	2.75 (0.64)	3.20 (0.62)	29.52 (2.81)
Medical insurance	No	3.38 (0.49)	2.89 (0.46)	3.07 (0.35)	3.22 (0.50)	2.77 (0.32)	3.18 (0.51)	3.16 (0.56)	3.25 (0.53)	3.09 (0.54)	3.03 (0.52)	30.32 (2.76)
	Yes	3.54 (0.57)^***^	2.89 (0.48)	3.18 (0.42)^***^	3.22 (0.60)	2.76 (0.39)	3.20 (0.59)	3.28 (0.53)^***^	3.27 (0.65)	3.12 (0.61)	3.20 (0.66)^***^	30.95 (3.40)^***^
District	Urban	3.34 (0.62)	2.57 (0.44)	3.13 (0.39)	3.07 (0.72)	2.82 (0.35)^***^	2.97 (0.67)	3.15 (0.56)	3.35 (0.62)	2.96 (0.64)	3.14 (0.61)	29.98 (3.21)
	Suburb	3.68 (0.43)^***^	3.06 (0.34)^***^	3.31 (0.31)^***^	3.32 (0.49)^***^	2.68 (0.41)	3.45 (0.36)^***^	3.45 (0.41)^***^	3.36 (0.59)^***^	3.26 (0.52)^***^	3.37 (0.63)^***^	32.12 (2.66)^***^
	Rural	3.5 (0.56)	3.02 (0.48)	3.05 (0.48)	3.25 (0.51)	2.78 (0.36)	3.17 (0.55)	3.17 (0.57)	3.11 (0.66)	3.1 (0.61)	2.99 (0.61)	30.36 (3.57)
**Health service utilization**											
Frequency of seeking health service in CHC	More than once per month	3.51 (0.57)	2.87 (0.48)	3.18 (0.42)^*^	3.23 (0.56)	2.76 (0.37)	3.19 (0.60)	3.26 (0.54)	3.28 (0.64)	3.09 (0.61)	3.21 (0.64)^***^	30.87 (3.35)
	Every 1–3 months	3.50 (0.56)	2.93 (0.51)	3.12 (0.40)	3.16 (0.65)	2.73 (0.39)	3.24 (0.50)	3.25 (0.52)	3.21 (0.67)	3.15 (0.61)	3.05 (0.64)	30.65 (3.42)
	More than every 3e months	3.50 (0.49)	2.96 (0.46)^*^	3.11 (0.37)	3.28 (0.61)	2.80 (0.39)	3.24 (0.51)	3.24 (0.53)	3.25 (0.58)	3.17 (0.55)	3.01 (0.61)	30.81 (3.04)
	Not sure	3.57 (0.52)	2.95 (0.41)	3.12 (0.40)	3.14 (0.62)	2.79 (0.36)	3.17 (0.50)	3.28 (0.48)	3.34 (0.51)	3.13 (0.53)	3.13 (0.63)	31.01 (2.71)
Times of	≤ 10	3.55 (0.52)	3.00 (0.45)^***^	3.13 (0.39)	3.30 (0.51)^*^	2.78 (0.39)	3.30 (0.47)^***^	3.31 (0.51)	3.25 (0.61)	3.21 (0.56)	3.11 (0.62)	3.16 (0.11)
outpatient visiting	10–14	3.60 (0.49)	2.98 (0.43)	3.24 (0.38)	3.22 (0.62)	2.75 (0.38)	3.21 (0.66)	3.32 (0.52)	3.37 (0.60)	3.15 (0.60)	3.23 (0.66)	3.15 (0.13)
in the previous	15–20	3.61 (0.52)^***^	2.84 (0.42)	3.30 (0.40)^***^	3.12 (0.72)	2.81 (0.32)^***^	3.26 (0.60)	3.43 (0.56)^***^	3.45 (0.59)^***^	3.24 (0.63)^***^	3.44 (0.60)^***^	3.40 (0.19)^***^
year	>20	3.33 (0.64)	2.69 (0.52)	3.06 (0.45)	3.19 (0.54)	2.72 (0.39)	3.03 (0.55)	3.05 (0.52)	3.10 (0.68)	2.89 (0.57)	3.04 (0.61)	3.11 (0.12)
Times of	0	3.51 (0.56)	2.90 (0.48)	3.16 (0.42)	3.23 (0.58)	2.76 (0.37)	3.21 (0.58)^*^	3.27 (0.54)^*^	3.28 (0.64)^*^	3.12 (0.61)^*^	3.18 (0.64)^***^	30.91 (3.36)
hospitalization in	1	3.53 (0.54)	2.81 (0.47)	3.13 (0.37)	3.20 (0.59)	2.75 (0.41)	3.11 (0.54)	3.15 (0.55)	3.17 (0.56)	3.01 (0.54)	3.07 (0.62)	30.20 (2.96)
the previous year	≥2	3.57 (0.49)^**^	2.99 (0.47)	3.23 (0.36)	3.28 (0.54)	2.71 (0.40)	3.21 (0.52)	3.23 (0.52)	3.22 (0.60)	3.10 (0.56)	2.99 (0.70)	30.71 (2.77)
Self-perceived	Fair/poor	3.47 (0.59)	2.86 (0.50)	3.12 (0.44)	3.27 (0.54)^***^	2.75 (0.38)	3.19 (0.56)	3.22 (0.55)	3.20 (0.65)	3.08 (0.58)	3.06 (0.62)	30.47 (3.32)
health status	Good/very good	3.57 (0.51)^***^	2.93 (0.44)^***^	3.22 (0.38)^***^	3.13 (0.63)	2.76 (0.38)	3.20 (0.59)	3.30 (0.52)^***^	3.36 (0.60)^***^	3.15 (0.62)^***^	3.31 (0.64)^***^	31.34 (3.21)^***^
Physical or mental	Yes	3.47 (0.62)	3.01 (0.47)^***^	3.21 (0.38)^***^	3.36 (0.51)^***^	2.75 (0.39)	3.38 (0.45)^***^	3.31 (0.50)^***^	3.23 (0.60)	3.19 (0.51)^***^	3.14 (0.63)	31.16 (3.02)^***^
disease lasting	No	3.52 (0.55)	2.87 (0.47)	3.16 (0.43)	3.14 (0.61)	2.77 (0.37)^***^	3.16 (0.59)	3.25 (0.55)	3.30 (0.66)^***^	3.10 (0.63)	3.20 (0.63)^***^	30.87 (3.42)
over 1 year	Not sure	3.57 (0.45)	2.74 (0.54)	3.03 (0.36)	3.33 (0.52)	2.67 (0.37)	3.02 (0.57)	3.13 (0.49)	3.11 (0.44)	3.01 (0.51)	2.92 (0.65)	29.64 (2.71)
Multimorbidity	Without	3.48 (0.56)	2.86 (0.47)	3.14 (0.43)	3.18 (0.60)	2.79 (0.35)	3.18 (0.59)	3.25 (0.54)	3.30 (0.64)	3.11 (0.61)	3.17 (0.63)	30.80 (3.31)
	With	3.54 (0.55)^**^	2.93 (0.49)^***^	3.20 (0.39)^***^	3.25 (0.56)	2.72 (0.41)^***^	3.22 (0.55)	3.27 (0.53)	3.23 (0.63)^**^	3.12 (0.60)	3.17 (0.65)	30.89 (3.30)
Total		3.51 (0.56)	2.89 (0.48)	3.16 (0.41)	3.22 (0.58)	2.76 (0.38)	3.20 (0.57)	3.26 (0.54)	3.27 (0.63)	3.11 (0.60)	3.17 (0.64)	30.84 (3.30)

* *P <0.05*;

** *P <0.01*;

*** *P <0.001*.

**Figure 1:**
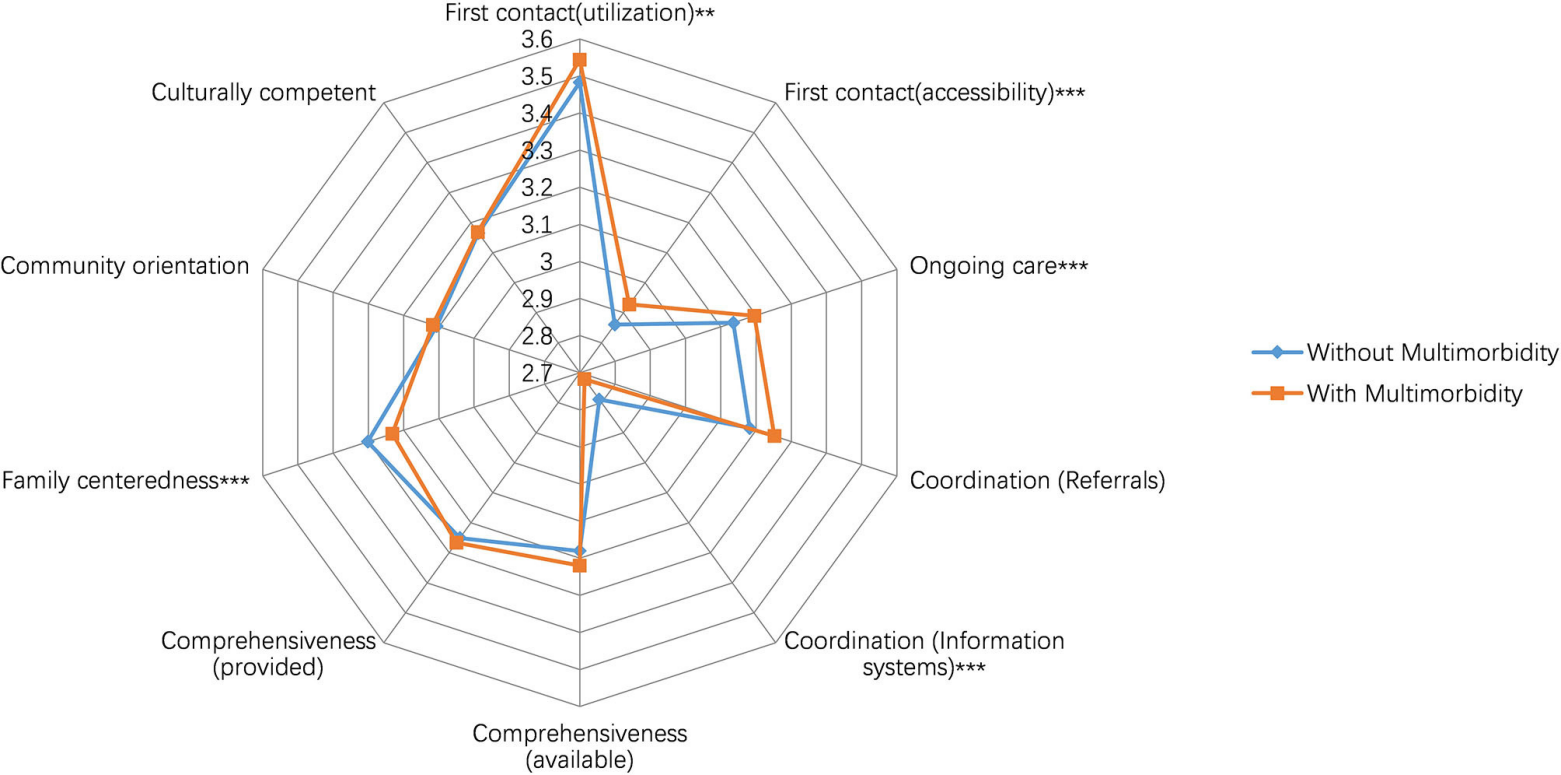
Scores of primary care attributes reported by participants with and without multimorbidity.

The radar chart shows the relationship between multimorbidity and primary care domain scores (Figure 1). The PCAT scores of each of the 10 domains are displayed for the two patient groups. The chart depicts the scores on a scale from 2.5 to 3.7 and gives an overall sense of each group's position. Patients with multimorbidity reported higher PCAT scores in the domains of first contact-utilization (3.54 ± 0.55 vs. 3.48 ± 0.56, *P* < 0.01), accessibility (2.93 ± 0.49 vs. 2.86 ± 0.47, *P* < 0.001), and ongoing care (3.20 ± 0.39 vs. 3.14 ± 0.43, *P* < 0.001). They reported lower scores in coordination (information system) (2.72 ± 0.41 vs. 2.79 ± 0.35, *P* < 0.001) and family-centeredness (3.23 ± 0.63 vs. 3.30 ± 0.64, *P* < 0.01). There were no significant differences between the two groups in the domains of coordination (referral system) (3.25 ± 0.56 vs. 3.18 ± 0.60), comprehensiveness (services available and provided) (3.22 ± 0.55 vs. 3.18 ± 0.59; 3.27 ± 0.53 vs. 3.25 ± 0.54, respectively), community orientation (3.12 ± 0.60 vs. 3.11 ± 0.61), and cultural competence (3.17 ± 0.65 vs. 3.17 ± 0.63).

### Multivariate Analyses of Primary Care Attributes

Table 3 presents the multi-variable linear regression results examining the association between multimorbidity and primary care quality scores, controlling for sociodemographic and health status characteristics. The multimorbid group had a higher total PCAT score (ß = 0.355, *P* < 0.01). Respondents who perceived higher total PCAT scores were also more likely to have a college education or above (ß = 0.826, *P* < 0.01), be located in a suburban area (ß = 1.475, *P* < 0.001), and have self-perceived good/excellent health status (ß = 0.337, *P* < 0.05). Older patients (61–70 years: ß = −0.623, *P* < 0.001; >70 years: ß = −0.573, *P* < 0.01), patients with an average monthly family income of ≥6,000 RMB (ß = −1.385, *P* < 0.001), patients with more than 20 outpatient visits in the previous year (ß = −1.883, *P* < 0.001), and patients reporting long lasting physical or mental disease (ß = −0.444, *P* < 0.01) had significantly lower total PCAT scores.

**Table 3:** Multiple regressions of participants'characteristics associated with individual and total primary care attributes scores.

**Variable**	**Group**	**First contact (utilization)**	**First contact (accessibility)**	**Ongoing care**	**Coordination (referral system)**	**Coordination (information system)**	**Comprehensiveness (available)**	**Comprehensiveness (provided)**	**Family centeredness**	**Community orientation**	**Culturally competent**	**Total**
		***B*-value**	***B*-value**	***B*-value**	***B*-value**	***B*-value**	***B*-value**	***B*-value**	***B*-value**	***B*-value**	***B*-value**	***B*-value**
Without multimorbidity		Reference										
With multimorbidity		0.076^***^	0.078^***^	0.07^***^	0.033	−0.031	0.035	0.051^*^	0.000	0.07^**^	0.009	0.355^**^
**Sociodemographic characteristics**												
Gender	Male	Reference										
	Female	0.001	−0.033	−0.009	−0.038	−0.036^*^	−0.006	−0.018	−0.023	−0.005	0.018	−0.142
Age (year)	≤ 60	Reference										
	61–70	−0.02	0.05^*^	−0.049^*^	−0.019	−0.07^***^	−0.108^***^	−0.092^***^	−0.076^*^	−0.101	−0.096^**^	−0.623^***^
	≥71	−0.006	0.025	−0.021	0.136^*^	−0.083^***^	−0.158^***^	−0.077^**^	−0.039	−0.07	−0.063	−0.573^**^
Marital Status	Married	Reference										
	Unmarried	−0.046	−0.031	−0.039	0.361	−0.146^*^	−0.131	−0.124	−0.053	−0.113	0.051	−0.715
Employment status	Employed	Reference										
	Unemployed/retired	0.012	−0.038	−0.030	−0.006	0.058^**^	0.122^***^	0.050	−0.086^**^	−0.013	0.129^***^	0.265
Education	Primary school or below	Reference										
	Junior school	−0.092^***^	0.057^*^	0.010	0.028	0.031	−0.068^*^	−0.044	0.009	−0.014	−0.117^***^	−0.212
	Senior high school	−0.082^*^	0.066^*^	−0.030	0.134^*^	0.029	−0.070	0.047	−0.033	0.005	−0.041	−0.09
	College or above	−0.045	0.100^***^	0.111^***^	0.371^***^	0.081^*^	0.054	0.163^***^	0.099	0.192^***^	0.016	0.826^**^
Average monthly	≤ 3,000	Reference										
household income	3,001–4,000	−0.058	−0.008	0.011	0.050	−0.026	0.040	−0.009	0.01	−0.009	0.039	−0.027
(RMB)	4,001–6,000	0.01	−0.027	−0.036	−0.053	−0.120^***^	0.028	−0.044	−0.087^*^	−0.023	−0.003	−0.366
	≥6,000	−0.033	−0.002	−0.075^*^	−0.338^***^	−0.154^***^	−0.185^***^	−0.195^***^	−0.344^***^	−0.246^***^	0.005	−1.385^***^
	Not sure	−0.049	−0.084^**^	−0.246^***^	−0.379^***^	−0.147^***^	−0.363^***^	−0.358^***^	−0.283^***^	−0.472^***^	−0.013	−2.110^***^
Medical insurance	No	Reference										
	Yes	0.105^***^	−0.034	0.073^***^	0.075	−0.007	−0.008	0.068^*^	−0.022	−0.023	0.103^**^	0.267
District	Urban	Reference										
	Suburb	0.228^***^	0.451^***^	0.109^***^	0.281^***^	−0.159^***^	0.424^***^	0.226	−0.122^***^	0.193^***^	0.167^***^	1.475^***^
	Rural	0.049	0.409^***^	−0.148^***^	0.082	−0.069^*^	0.147^***^	−0.054	−0.443^***^	−0.023	−0.062	−0.284
**Health care utilization**												
Frequency of seeking health service in CHC	More than once per month	Reference										
	Every 1–3 months	−0.09^**^	−0.049	−0.056^*^	−0.178^**^	−0.055^*^	−0.058	−0.105^**^	−0.085	−0.072	−0.189^***^	−0.786^***^
	More than every 3 months	−0.084^*^	−0.056	−0.064^*^	−0.012	−0.005	−0.055	−0.111^**^	−0.052	−0.06	−0.227^**^	−0.686^**^
	Not sure	0.016	−0.023	−0.001	−0.149	0.000	−0.055	−0.007	0.091	−0.016	−0.052	−0.029
Times of	≤ 10	Reference										
outpatient visiting	10–14	−0.019	−0.026	0.044	−0.100	−0.038	−0.115^***^	−0.050	0.056	−0.106^**^	−0.012	−0.281
in the previous	15–20	−0.021	−0.124^***^	0.07^*^	−0.168	0.025	−0.099^**^	0.009	0.072	−0.047	0.136^**^	0.087
year	>20	−0.229^***^	−0.219^***^	−0.116^***^	−0.093	−0.091^***^	−0.226^***^	−0.293^***^	−0.214^***^	−0.332^***^	−0.139^***^	−1.883^***^
	≥2	0.08	0.059	0.109^*^	0.042	0.011	0.006	0.034	0.107	0.048	−0.094	0.346
Self–perceived	Fair/poor	Reference										
health status	Good/very good	0.03	0.017	0.054^**^	−0.07	0.019	−0.069^**^	−0.002	0.113^***^	0.025	0.154^***^	0.337^*^
Physical or mental	Yes	Reference										
disease lasting	No	0.034	−0.112^***^	−0.045^*^	−0.129^**^	−0.006	−0.188^***^	−0.068^**^	−0.007	−0.110^***^	0.012	−0.444^**^
over 1 year	Not sure	0.094^*^	−0.33^***^	−0.147^***^	−0.027	−0.096^**^	−0.367^***^	−0.184^***^	−0.126^*^	−0.218^***^	−0.137^**^	−1.535^***^
	Adjusted *R*^2^	0.098	0.272	0.149	0.145	0.067	0.218	0.164	0.117	0.145	0.128	0.189

* *P <0.05*;

** *P <0.01*;

*** *P <0.001*.

## Discussion

We assessed the quality of primary care in Shanghai from the perspective of patients using a validated Chinese version of the PCAT-AE. Overall, respondents in our study reported lower PCAT scores than patients from CHCs in the US. This might be due to our exclusion of the coordination (referral system) domain in our scoring (42). It may also be due to China's still under-developed primary healthcare system, especially in comparison with that of developed countries. Total PCAT scores vary between studies of different areas of China because of differences in sample selection and the survey tool used. The total PCAT score was a little lower than that of a study conducted in the Guangdong Province (43) which included all CHC users regardless of their usual source of care. Our study focused on contracted residents who more frequently utilized both medical and health management services provided by CHCs. The Guangdong study also used an abbreviated version of the PCAT with only 25 items used to assess seven domains of primary care, whereas the PCAT-AE contains 87 items to assess 10 domains of primary care. However, in comparison with previous studies conducted in Shanghai in 2013, this study's total PCAT score was higher (25). This suggests that medical reform measures may effectively improve the quality of primary care.

Although no significant difference existed between the total PCAT scores for the groups with and without multimorbidity, there were disparities between the different PCAT domains. Our results revealed that patients with multimorbidity reported higher scores in first contact (utilization and accessibility) and ongoing care. This may be due to the establishment of CHCs as the preferred primary care providers in Shanghai or the accessibility of CHCs (i.e., every resident can reach a CHC on foot within 15 min). Additionally, since the implementation of the General Practice Residents training policy, Shanghai has achieved its goal of having one GP for every 3.5 million residents. These measures make it easier for residents to access a healthcare provider in Shanghai. The family physician contracted policy also helps establish long-term relationships between patients and doctors. The convenient service provided by CHCs promotes care equity (25) and continuity of services (44). These results show the advantages of GP and primary care services in promoting care continuity (45).

However, the results also showed that scores on coordination (information system) were lower among patients with multimorbidity compared to those without multimorbidity. A lower score could be explained by the fact that the referral system is still imperfect in China. In our study, only 851 contracted patients reported experiencing a referral. Chinese patients have the freedom to choose a doctor across all three levels of hospitals. This weakens the referral coordination attribute of primary care. In addition, the referral information systems between hospitals need improvement. Although the Shanghai Municipal Government has assisted in building and updated the outpatient and emergency treatment information database of all hospital levels in Shanghai, the information systems have yet to be interconnected across healthcare organizations. China should consider strengthening coordination between primary healthcare institutions and hospitals while simultaneously modernizing its primary healthcare system through the establishment of a learning health system built on digital data and innovative technologies (46).

In addition to coordination (information system), the multimorbid group reported lower scores for the family-centeredness domain. Providers treating complex diseases can benefit from consulting a patient's family members and shared in decision-making, especially in the case of aging or multimorbid patients. GPs should pay attention to the utilization of family resources in the diagnosis and treatment of multimorbidity.

There were no differences between the two groups' PCAT scores in the domains of comprehensiveness (services available and provided), community orientation, and cultural competence. The likely reason for this result is that basic public health services are fully implemented in CHCs in Shanghai; for example, periodic health assessments, home care services and traditional Chinese medicine are all available and utilized, with an emphasis on early detection and follow-ups (47). Meanwhile, GP teams have a good recognition of community health needs and adapt to the cultural specificities of the community they serve.

As for demographics, our results indicated that respondents who were older and in relatively good health reported higher total PCAT scores. This is consistent with a Korean study based on a data sample collected from patients whose usual source of care came from family doctors working at nine private clinics. The Korean PCAT found that primary care quality was positively associated with good self-rated health status (48). Our study also found that those with an education of college or above and higher average income would report significantly lower total PCAT scores. This may be caused by participants in these groups being more inclined to seek out higher-level hospitals for care. A previous study in China found that compared with other types of healthcare facilities, tertiary hospital users had greater proportions of patients with higher education, employment, and income levels (43).

Our study had several limitations. First, the participants in our study were limited to family practice contracted patients only. Due to the pilot nature of the program, our results may not be as generalizable for other regions. Second, recall bias might have occurred during administration of the questionnaire, although we screened patients to make sure they visited at least twice in the previous half year. Third, in order to acquire sufficient data from each patient, those with cognitive impairment who couldn't understand the questions or who were unable to complete the questionnaire were excluded from this survey. Although these patients accounted for a small proportion in the outpatient population of community health centers, their exclusion might have introduced sampling bias. Finally, because multimorbidity was defined by the patient's subjective report of disease history and not by the doctor's medical history diagnosis, this might also have been subject to recall bias. In addition, we did not group patients with different disease patterns or severity, so it was possible that different combinations or severities of disease might have impacted patient experiences. Further research is needed to investigate these issues.

## Conclusion

Our study assessed primary care quality based on patient experiences at CHCs in Shanghai, China. Patients with multimorbidity had better experiences in the domains of first contact-utilization, accessibility, and ongoing care compared to patients without multiple diseases. Still, deficiencies in the coordination (information system) and family-centeredness domains persist. Our findings provide a reference for policy development and medical reform in Shanghai. It is necessary to strengthen policy to promote the implementation of a two-way referral system and integrate and improve the medical and health information system. General practitioners should pay more attention to the needs of patients with multimorbidity, especially regarding family-centeredness, so as to promote better patient satisfaction.

## Data Availability Statement

The original contributions presented in the study are included in the article/supplementary material, further inquiries can be directed to the corresponding author/s.

## Ethics Statement

The studies involving human participants were reviewed and approved by Ethics Committees of Tongji University (ref: LL-2016-ZRKX-017). The patients/participants provided their written informed consent to participate in this study.

## Author Contributions

HJ, LS, and DY conceived and designed the study. JS, ZW, and CC analyzed the data. YH, WH, YL, YZ, and XG contributed reagents, materials, and analysis tools. HJ and JS wrote paper. All authors have read and approved the manuscript.

## Conflict of Interest

The authors declare that the research was conducted in the absence of any commercial or financial relationships that could be construed as a potential conflict of interest.

## Abbreviations

CHCs: Community Health Centers
GPs: General Practitioners
PCAT: Primary Care Assessment Tool
PCAT-AE: Primary Care Assessment Tool-Adult Edition.
